# Intake of n-3 Polyunsaturated Fatty Acids Increases Omega-3 Index in Aged Male and Female Spontaneously Hypertensive Rats

**DOI:** 10.5402/2013/209360

**Published:** 2013-02-19

**Authors:** Barbara Bačová, Peter Seč, Milan Čertik, Narcis Tribulova

**Affiliations:** ^1^Institute for Heart Research, Slovak Academy of Sciences, Dúbravská Cesta 9, P.O. Box 104, 840 05 Bratislava, Slovakia; ^2^Institute of Animal Biochemistry and Genetics, Slovak Academy of Sciences, Bratislava, Slovakia; ^3^Faculty of Chemical and Food Technology, Slovak University of Technology, Bratislava, Slovakia

## Abstract

The purpose of this study was to examine whether n-3 PUFA intake affects n-3 and n-6 FA levels in plasma and red blood cells as well as omega-3 index in old male and female spontaneously hypertensive (SHR) and healthy rats. Plasma linoleic acid and eicosapentaenoic acid increased due to n-3 PUFA intake in SHR and healthy rats. Comparing to healthy rats the levels of PUFA in red blood cells of SHR were lower in males and higher in females with exception of arachidonic acid, which was high in males and low in females. Feeding of rats with n-3 PUFA resulted in increase of red blood cells levels of eicosapentaenoic acid and/or docosahexaenoic acid in a sex- and strain-dependent manner. Moreover, n-3 PUFA intake decreased arachidonic acid in healthy female rats but increased it in SHR and did not affect it in males. Omega-3 index was lower in SHR comparing to healthy rats and it increased due to the consumption of n-3 PUFA. Results point out sex- and strain-related differences in red blood cells levels of n-3 and n-6 PUFA in basal conditions as well as in response to n-3 PUFA intake.

## 1. Background

Polyunsaturated n-3 fatty acids (n-3 PUFA) are important components of cell membranes affecting their function, as they are incorporated into the phospholipids. Dietary deprivation of the essential fatty acids is deleterious to health. Mammals do not produce Δ12 and Δ15 desaturase enzymes and therefore cannot produce either linoleic acid (LA; 18:2n-6) or *α*-linolenic acid (ALA; 18:3n-3), precursors of the n-6 and n-3 fatty acids de novo [1]. ALA is metabolized to produce long-chain n-3 (omega) PUFA, the most prevalent of which are eicosapentaenoic acid (EPA; 20:5n-3) and docosahexaenoic acid (DHA; 22:6n-3). LA is metabolized to produce longchain n-6 PUFA, such as arachidonic acid (AA; 20:4n-6) and gamma-linolenic acid (GLA; 18:3n-6). EPA is further metabolized by the cyclooxygenase and lipoxygenase pathways to produce the n-3 series of eicosanoids (prostaglandins, thromboxanes, and leukotrienes) that are potent, short-acting, local hormones, or second messengers exhibiting anti-inflammatory properties, while the n-2 series of eicosanoids derived from AA are rather proinflammatory [1]. A reduced ratio AA/EPA shifts the spectrum of ecosanoids production toward an increase in thromboxane A_3_ and PGI_3_ at the expense of TXA_2_ and PGI_2_. This shift was found to reduce the risk of fatal arrhythmias [2].

Data from clinical [3, 4] and experimental studies [5, 6] support the hypothesis that consumption of n-3 PUFA lowers the risk of cardiovascular diseases and sudden cardiac death [4, 7]. Based on the scientific evidence a new risk factor for sudden cardiac death was proposed, the omega-3 index [7]. It is measured in red blood cells and is expressed as a percentage of EPA + DHA of total fatty acids. Red blood cells fatty acid composition reflects long-term intake of EPA + DHA and tissue fatty acids composition, including the cardiac muscle [8]. Hypertension is one of the main risk factors for cardiovascular diseases and we have previously shown [5, 9, 10] that the hypertensive rats benefit from n-3 PUFA intake due to protection from life-threatening arrhythmias. These antiarrhythmic properties were associated with treatment-induced improvement of cell membrane and intercellular gap junction integrity linked with upregulation of myocardial electrical coupling protein, connexin-43. Thus, we aimed to explore whether pure n-3 PUFA intake affects plasma and particularly red blood cells profile of n-3 and n-6 PUFA in aged male and female hypertensive rats that exhibit connexin-43 abnormalities and are prone to lethal arrhythmias comparing to age-matched healthy rats.

## 2. Methods

All animal experiments were performed in accordance with the rules issued by the State Veterinary Administration of the Slovak Republic, Legislation no. 289/2003 and they conform to the European Convention for the Protection of Vertebrate Animals used for Experimental and other Scientific Purposes (Council of Europe no. 123, Strasbourg 1985). Experiments were conducted on spontaneously hypertensive rat (SHR), a rodent model that mimics human essential hypertension and is suitable to investigate hypertension-induced myocardial changes and transition from compensated left ventricular hypertrophy to heart failure in aging (>1 year-old rats). Male, 12-month-old SHRs as well as their age-matched healthy Wistar rats were used in this study. Animals were divided into two groups: untreated rats (*n* = 30) fed by standard laboratory chow and rats (*n* = 30) supplemented with pure omega-3 ethyl ester (EE: 65% DHA + EPA, Vesteraalens, Norway, 200 mg/kg b.w./day) for two months.

### 2.1. Animal Monitoring and Tissue Sampling

Blood pressure was measured by tail-cuff plethysmography using the Statham Pressure Transducer P23XL (Hugo Sachs, Germany) and monitored together with body weight at the beginning and the end of the experiments. Fasting blood glucose was measured by using blood glucose test meter. The hearts from the anaesthetized rats were rapidly excised into ice-cold saline to arrest heart beat and the weight of the heart and left ventricle was registered. Blood from the heart was drawn into ethylenediaminetetraacetic acid tubes and centrifuged at 4°C to separate cells from plasma. The buffy coat was removed and plasma and red blood cells were frozen in liquid nitrogen and kept at −80°C until analyzed for fatty acids composition by gas chromatography.

### 2.2. Lipid Extraction and Fatty Acids Analysis

Total lipids from plasma and red blood cells samples were extracted three times with chloroform containing internal standard of heptadecanoic acid (Supelco, USA). Collected chloroform layers were evaporated under vacuum and lipids were dissolved in hexane/chloroform (9 : 1, v/v). Fatty acids from total lipids were converted to their methylesters by methanolic solution of sodium methoxide and methanolic HCl and analysed by gas chromatography (GC-6890 N, Agilent Technologies) using a capillary column DB-23 and a FID detector as previously described [11]. The fatty acid methylester peaks were identified by authentic standards of C_4_–C_24_ fatty acid methylesters mixture (Supelco, USA) and evaluated by ChemStation B 01 03 (Agilent Technologies). To identify the unknown peaks in lipid structures, GC-MS analysis of fatty acid methyl esters was also performed. All peaks were evaluated by MSD ChemStation E.00.00.202 with combination of NIST Mass Spectral Search Program version 2.0 d (Agilent Technologies). The level of the following n-3 PUFAs: alfa linolenic acid (ALA), eicosapentanoic acid (EPA), and docosahexanoic acid (DHA), as well as n-6 PUFA: linoleic acid (LA), arachidonic acid (AA), and gamma-linolenic acid (GLA) was expressed in percentage of total free fatty acids. Omega-3 index was expressed as a percentage of EPA + DHA of total fatty acids measured in red blood cells.

### 2.3. Statistical Analysis

The data are expressed as means ± standard deviations (SD). One-way ANOVA followed by Newmann-Keuls test was used to analyze the statistical significance between means. Comparison between the two groups was performed using the two-tailed Student's *t*-test. Categorical variables were compared using the Fisher exact test or chi-squared test. Values were considered to differ significantly at *P* < 0.05.

## 3. Results

### 3.1. Main Characteristics of Experimental Rats

Both male and female hypertensive rats exhibited significantly higher systolic blood pressure as well as heart and left ventricular weights compared to nonhypertensive rats. Body weight of male and female hypertensive rats was lower comparing to age-matched Wistar rats. Furthermore, male hypertensive rats exhibited significantly lower levels of blood glucose. Consumption of n-3 PUFA reduced significantly blood pressure in female but not in male hypertensive rats and reduced blood glucose in male but increased it in female hypertensive rats. Moreover, n-3 PUFA supplementation reduced left ventricular weight in both male and female hypertensive rats and decreased body weight in male Wistar rats but increased it in females of the same strain. Data are summarized in Tables 1 and 2.

### 3.2. Fatty Acids Levels in Plasma

**Figure 1:**
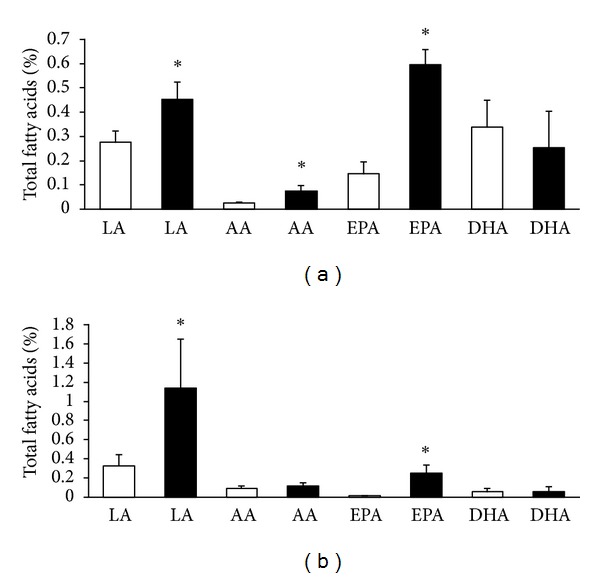
Plasma n-6 PUFA and n-3 PUFA composition in male Wistar rats (a) and SHR (b); LA: linolenic acid, AA: arachidonic acid, EPA: eicosapentaeneic acid, DHA: docosahexaenoic acid, untreated rats: white columns, and n-3 PUFA-treated rats: black columns. Values are means ± SD of 15 rats in each group. Significant difference from untreated rats: *P < 0.05.

**Figure 2:**
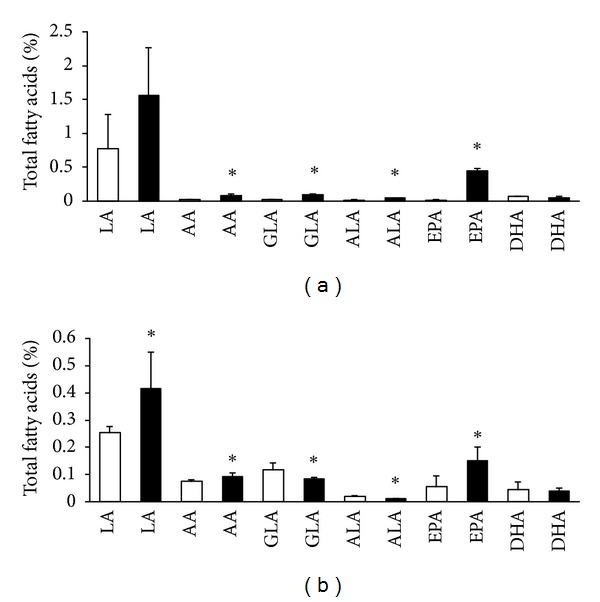
Plasma n-6 PUFA and n-3 PUFA composition in female Wistar rats (a) and SHR (b); LA: linolenic acid, AA: arachidonic acid, GLA: gamma-linoleic acid, ALA: alfa linolenic acid, EPA: eicosapentaeneic acid, DHA: docosahexaenoic acid, untreated rats: white columns, and n-3 PUFA-treated rats: black columns. Values are means ± SD of 15 rats in each group. Significant difference from untreated rats: *P < 0.05.

Comparing to normotensive rats the circulating levels of EPA and DHA in plasma were lower in male but not in female SHR (Figures 1(a), 1(b), 2(a), and 2(b)). There was no apparent difference in the level of LA between males Wistar and SHR unlike females in which LA was lower in SHR than Wistar rats. Intake of n-3 PUFA resulted in significant increase of EPA but not DHA in plasma of Wistar and SHR regardless of the sex. Moreover, treatment led to a significant increase of LA in male as well as female SHR and Wistar rats.

### 3.3. Fatty Acids Levels in Red Blood Cells

**Figure 3:**
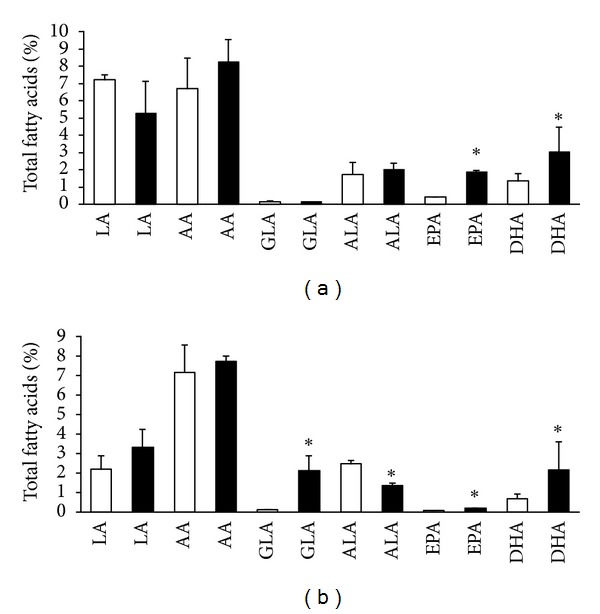
Red blood cell n-6 PUFA and n-3 PUFA composition in male Wistar rats (a) and SHR (b); LA: linolenic acid, AA: arachidonic acid, GLA: gamma-lonoleic acid, ALA: alfa linolenic acid, EPA: eicosapentaeneic acid, DHA: docosahexaenoic acid; untreated rats: white columns, and n-3 PUFA-treated rats: black columns. Values are means ± SD of 15 rats in each group. Significant difference from untreated rats: *P < 0.05.

**Figure 4:**
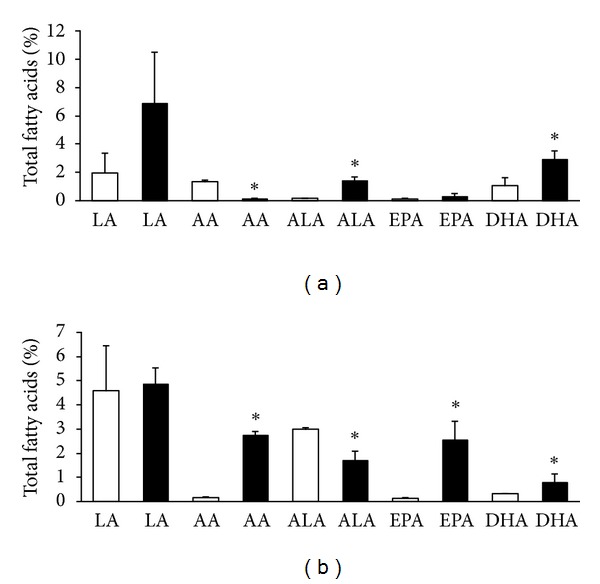
Red blood cell n-6 PUFA and n-3 PUFA composition in female Wistar rats (a) and SHR (b); LA: linolenic acid, AA: arachidonic acid, ALA: alfa linolenic acid, EPA: eicosapentaeneic acid, DHA: docosahexaenoic acid, and untreated rats: white columns, and n-3 PUFA-treated rats: black columns. Values are means ± SD of 15 rats in each group. Significant difference from untreated rats: *P < 0.05.

Comparing to normotensive rats the red blood cells levels of EPA and particularly DHA were lower in both male and female SHR (Figures 3(a), 3(b), 4(a), and 4(b)). AA was almost six times higher in males than females either SHR or Wistar rats. On the other hand, LA level was lower in male but higher in female SHR compared to Wistar rats. Intake of n-3 PUFA resulted in significant increase of EPA as well as DHA in both sexes of SHR and Wistar rats. The increase of EPA in female Wistar rats was, however, not significant. Furthermore, the treatment was accompanied by reduction of ALA in male as well as female SHR but not in Wistar rats. Feeding of rats with n-3 PUFA did not affect significantly either LA or AA in males, either SHR or Wistar rats, but increased AA in female SHR and suppressed it in Wistar rats.

Omega-3 index was lower in both male and female SHR comparing to age-matched Wistar rats but significantly increased due to n-3 PUFA intake in both strains regardless of the sex. Moreover, treatment was associated with a decrease in AA/EPA ratio in male as well as female SHR and Wistar rats. However, the ratio n-6 : n-3 PUFA was decreased in Wistar rats only and not in n-3 PUFA feed SHR (Tables 3 and 4).

## 4. Discussion

We have found in this study that there are strain- and sex-dependent differences in n-3 and n-6 PUFA profile in plasma and red blood cells of aged healthy and hypertensive rats. In general, there are low levels of PUFA (with exception of LA) in plasma of females Wistar or SHR when compared to males. It may indicate that the rate of PUFA metabolism is faster in females even if they are old. Women also have a greater capacity than man to synthesise n-3 PUFA from their essential fatty acid precursor ALA [12]. Moreover, turnover of n-3 PUFA in plasma of old males decreased with age [13]. Furthermore, we have found strain- and sex-related differences in red blood cells levels of ALA, which was almost three times higher in female SHR than age-matched Wistar rats and comparing to males. In contrast, red blood cells levels of AA were much higher in males SHR and Wistar rats than females but its level in plasma was low regardless the sex or strain. Sex-related differences point out the role of sex hormones in regulation of PUFA metabolism. It has been hypothesised that sex differences are established in order to ensure adequate supply of PUFA to the developing foetus [14]. On the other hand, there were no strain- and sex-dependent differences in the level of LA, which was high in comparing to other PUFAs either in plasma or red blood cells of all examined rats. The similar feature of plasma profile of PUFA as revealed in male Wistar rats (including higher concentration of LA) has been found by others in young or middle-aged healthy male rats [15–17]. However, the data in old animals are missing. In experimental studies, LA has been shown to reduce the serum levels of low-density lipoprotein cholesterol, especially when substituted for saturated fatty acids [18]. It has been reported that the competition of LA with n-3 and n-6 PUFAs for phospholipid incorporation may account for, the significant inverse correlation of LA with EPA, DHA, and AA shown in red blood cells of humans [19]. Perhaps this is a case of male as well as female SHR and Wistar rats that exhibited relatively low red blood cells levels of EPA, DHA, and AA (the latter in females only). LA also competes with ALA for a single set of desaturating and elongating enzymes and the relative levels of n-3 and n-6 PUFA in animal tissues can be regulated by altering the balance between LA and ALA. Dietary LA is known to suppress DHA synthesis [20]. Nevertheless, likewise healthy humans [21] the levels of DHA in plasma or red blood cells were higher than EPA in Wistar rats but not in SHR. It should be noted that n-3 and n-6 PUFA profile found in plasma reflects short-term changes, whereas in red blood cells rather long-term changes in dietary fatty acids intake.

The main results of our study point out that two-month-lasting supplementation with pure n-3 PUFA resulted in significant increase of both plasma and red blood cells levels of EPA and/or DHA in healthy and particularly hypertensive rats. An increase of EPA and DHA was accompanied by reduction in ALA levels most likely due to its enhanced metabolism. Moreover, there was apparent strain- and sex-dependent difference in treatment response to red blood cells fatty acids profile. Increase of EPA in males was higher in Wistar rats comparing to SHR while this relationship was opposite in females. Noteworthy, AA was not changed due to n-3 PUFA intake in red blood cells of males unlike the females in which its level was significantly increased in SHR but suppressed in Wistar rats (decrease was also shown by others [17]). Interestingly, n-3 PUFA-induced increase of AA was associated with significant decrease of the blood pressure in females SHR. However, blood pressure was not affected in male SHR in which AA did not change. It may indicate the implication of AA in modulation of vascular resistance. In this context the dual role of AA in biological processes should be noted. Not all eicosanoids derived from AA are proinflammatory or adverse for vascular function. Indeed, AA stimulates production of prostacyclin (PGI_2_) by vascular tissue, a compound that exhibits antithrombotic effects and intake of n-3 PUFA enhanced it [22]. Moreover, AA is converted to epoxyeicosatrienoic acids (EETs) by epoxygenases or to 20-hydroxyeicosatetraeonic acid (20-HETE) by omega-hydrolases [23]. 20-HETE, a vasoconstrictor associated with endothelial dysfunction, is higher in hypertensive individuals as well as SHR and androgens increase its synthesis [24]. Thus, it appears that males might be more prone to hypertension than females. On the other hand, EETs are vasodilators and anti-inflammatory mediators. They are antihypertensive, since their intravenous infusion significantly reduced blood pressure in SHR [25]. Red blood cells represent reservoirs and carriers of EETs in the circulation. Importantly, red blood cells in addition to O_2_ delivery in the microvasculature participate in the regulation of microvascular tone by releasing vasoactive factors, including EETs [26]. Supplementation of rats with n-3 PUFA also affected glucose metabolism by sex-dependent manner, that is, decreased blood glucose levels in males Wistar and SHR but increase it in females of both groups.

Our study revealed for the first time that omega-3 index is low in male and female SHR but it increased due to n-3 PUFA intake. It was associated with significant decrease of blood pressure in male but not female SHR suggesting sex differences in modulation of cardiovascular disease risk factors, whereby sex-related differences in AA metabolism can be involved (pointed above). In this context it should be emphasised, the inverse association of omega-3 index with inflammatory markers, such as C-reactive protein and interleukin-6 that was observed in humans [27]. It can be expected that n-3 PUFA intake may suppress the process of inflammation in SHR as well. Because red blood cells reflect heart tissue PUFA compositions we can assume that increase in omega-3 index is associated with enhanced EPA and DHA incorporation into cardiomyocyte cell membranes. This may contribute to improvement of cell membrane and gap junction integrity demonstrated previously in aged male and female SHR heart [5] and consequently affecting ion and connexin-43 channels function to suppress arrhythmogenesis.

## Figures and Tables

**Table 1:** General characteristics of male SHR and Wistar rats.

	WRc	WRn3	SHRc	SHRn3
BP (mmHg)	112 ± 6	94 ± 10*	177 ± 3*	174 ± 9
BW (g)	473 ± 48	442 ± 22*	342 ± 7*	343 ± 16
HW (g)	1.19 ± 0.10	1.13 ± 0.07	1.41 ± 0.04*	1.40 ± 0.08
LVW (g)	0.85 ± 0.03	0.82 ± 0.05	1.16 ± 0.05*	1.11 ± 0.08^#^
HW/BW (mg/g)	2.53 ± 0.22	2.55 ± 0.11	4.12 ± 0.17*	4.08 ± 0.23
LVW/BW (mg/g)	1.81 ± 0.18	1.86 ± 0.17	3.40 ± 0.16*	3.25 ± 0.22^#^
BG (mmol/L)	5.35 ± 0.41	3.86 ± 0.29*	5.03 ± 0.10*	4.53 ± 0.46^#^

Values are means ± SD of 15 rats in each group. WRc: Wistar control rats, WRn3: Wistar rats fed with omega-3 fatty acid, SHRc: SHR control rats, SHRn3: SHR rats fed with omega-3 fatty acid, BP: blood pressure, BW: body weight, HW: heart weight, and LVW: left ventricular weight. Significant difference from WISc: *P < 0.05. Significant difference from SHRc: ^#^ *P* < 0.05.

**Table 2:** General characteristics of female SHR and Wistar rats.

	WRc	WRn3	SHRc	SHRn3
BP (mmHg)	101 ± 4	99 ± 8	200 ± 16*	162 ± 28^#^
BW (g)	279 ± 24	299 ± 29*	231 ± 21*	217 ± 11^#^
HW (g)	0.83 ± 0.06	0.85 ± 0.04	1.31 ± 0.12*	1.16 ± 0.16^#^
LVW (g)	0.60 ± 0.03	0.61 ± 0.05	1.04 ± 0.10*	0.90 ± 0.13^#^
HW/BW (mg/g)	2.99 ± 0.38	2.87 ± 0.33	5.26 ± 1.08*	5.34 ± 0.63
LVW/BW (mg/g)	2.16 ± 0.25	2.08 ± 0.25	4.16 ± 0.81*	4.13 ± 0.51
BG (mmol/L)	4.05 ± 0.55	4.35 ± 0.73	4.00 ± 0.94	5.34 ± 0.87^#^

Values are means ± SD of 15 rats in each group. WRc: Wistar control rats, WRn3: Wistar rats fed with omega-3 fatty acid, SHRc: SHR control rats, SHRn3: SHR rats fed with omega-3 fatty acid, BP: blood pressure, BW: body weight, HW: heart weight, and LVW: left ventricular weight. Significant difference from WISc: *P < 0.05. Significant difference from SHRc: ^#^ *P* < 0.05.

**Table 3:** The levels of n-3 PUFA and n-6 PUFA in red blood cells of male SHR and Wistar rats.

	WRc	WRn3	SHRc	SHRn3
n-6 PUFA (%)	14.03	13.62	9.48	13.15
n-3 PUFA (%)	3.46	6.87	3.21	3.72
n-6/n-3 PUFA	3/1	2/1	2/1	4/1
EPA + DHA	1.75	4.88	0.73	2.38
AA/EPA	16.72	4.41	110.12	40.58

WRc: Wistar control rats, WRn3: Wistar rats fed with n-3 PUFA, SHRc: SHR control rats, and SHRn3: SHR rats fed with n-3 PUFA.

**Table 4:** The levels of n-3 PUFA and n-6 PUFA in red blood cells of female SHR and Wistar rats.

	WRc	WRn3	SHRc	SHRn3
n-6 PUFA (%)	3.33	7.01	4.74	7.58
n-3 PUFA (%)	1.31	4.54	3.43	5.03
n-6/n-3 PUFA	3/1	2/1	1/1	2/1
EPA + DHA	1.17	3.17	0.44	3.34
AA/EPA	10.5	0.45	1.25	1.07

WRc: Wistar control rats, WRn3: Wistar rats fed with n-3 PUFA, SHRc: SHR control rats, and SHRn3: SHR rats fed with n-3 PUFA.
